# Supplementary material for: A Fenton-Like Nanocatalyst Based on Easily Separated Magnetic Nanorings for Oxidation and Degradation of Dye Pollutant
Source: Materials (Basel). 2020 Jan 11;13(2):332. doi: 10.3390/ma13020332 (PMC7014477; doi:10.3390/ma13020332)
Supplement: Supplementary file 1 [file materials-13-00332-s001.pdf]

materials

Supporting Information

A Fenton-Like Nanocatalyst Based on Easily Separated Magnetic Nanorings for Oxidation and Degradation of Dye Pollutant

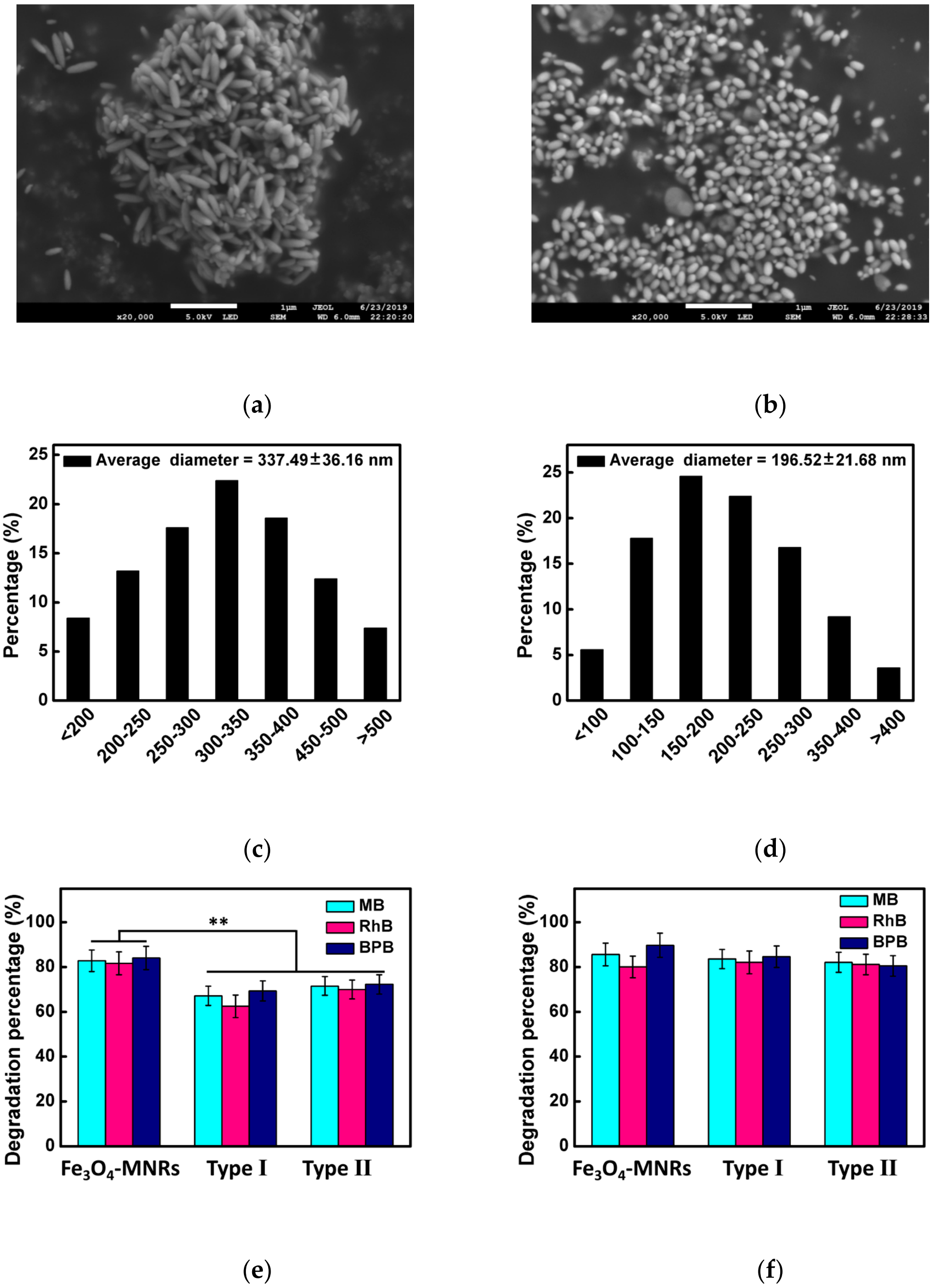

Figure S1. SEM images (**a**,**b**) and size distribution (**c**,**d**) of the Type I and Type II Fe_3O_4 nanoparticles. (**e**) Dyes (MB, RhB and BPB) degradation efficiency by different Fe_3O_4 nanoparticles (240 min, 20 °C, pH 5.0). (**f**) Reusability of different Fe_3O_4 nanoparticles after 10 cycles reuse.

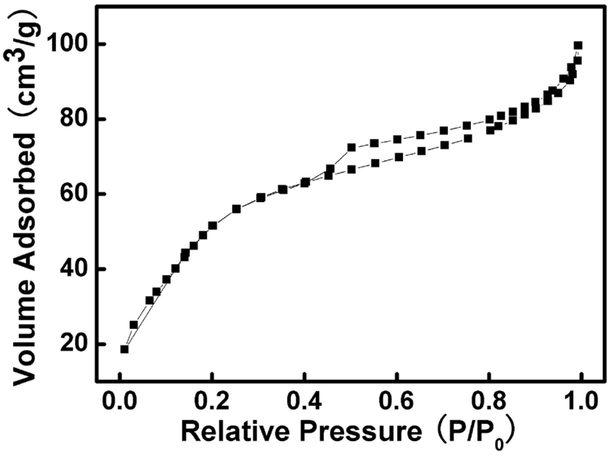

Figure S2. N_2 adsorption-desorption isotherms of the as-synthesized Fe_3O_4-MNRs.

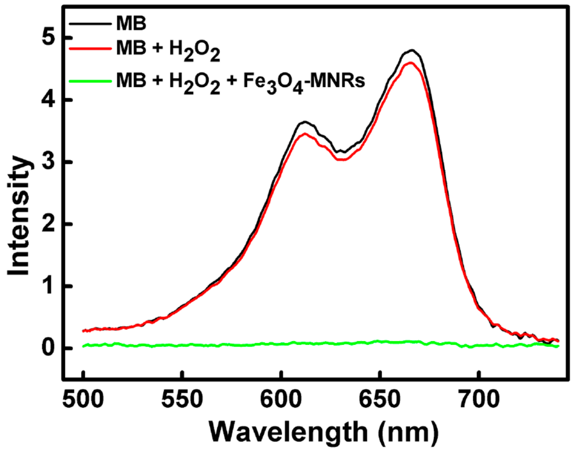

(**a**)

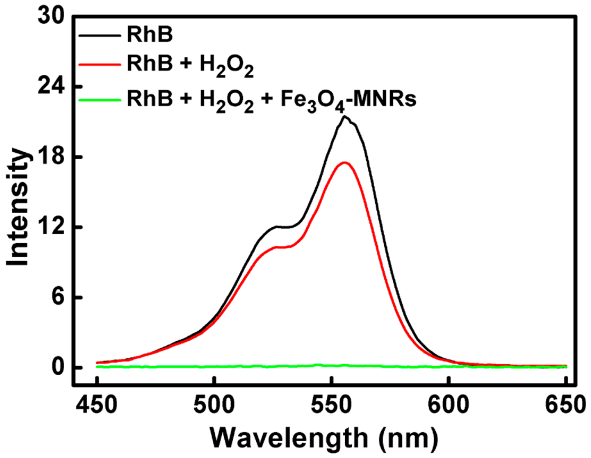

(b)

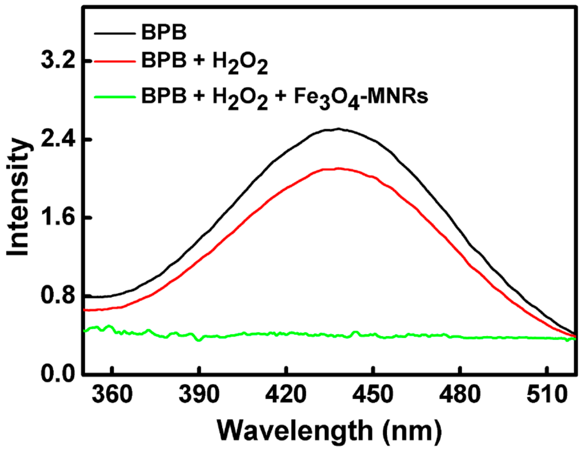

(c)

Figure S3. UV-vis curves of MB (**a**), RhB (**b**) and BPB (**c**) solution treated with or without Fe_3O_4-MNRs after 2 day treatment.

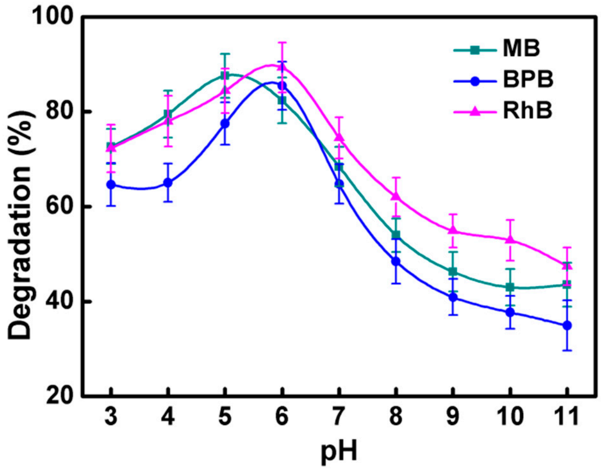

Figure S4. Dyes degradation efficiency of Fe_3O_4-MNRs with pH ranges from 3.0 to 11.0.

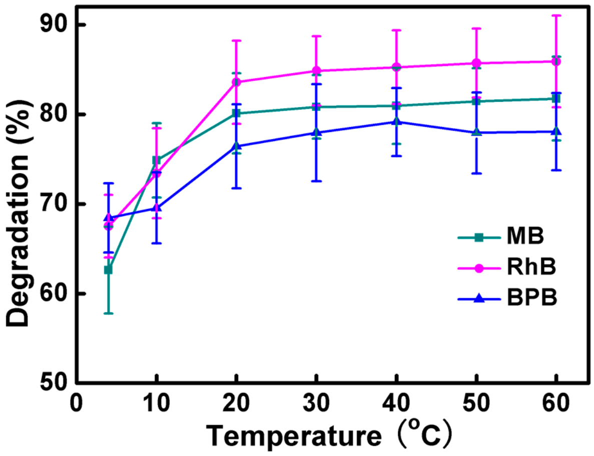

Figure S5. Dyes degradation efficiency of Fe_3O_4-MNRs at different temperature.

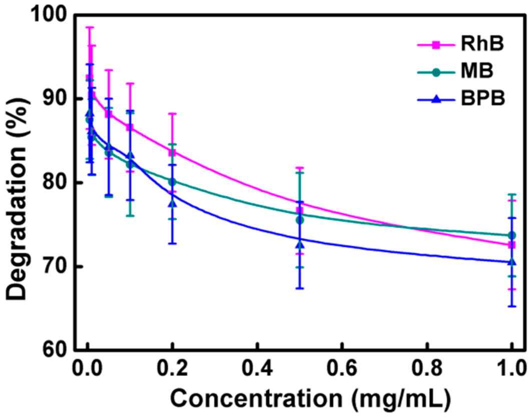

Figure S6. Dyes degradation efficiency of Fe_3O_4-MNRs with various initial concentration.

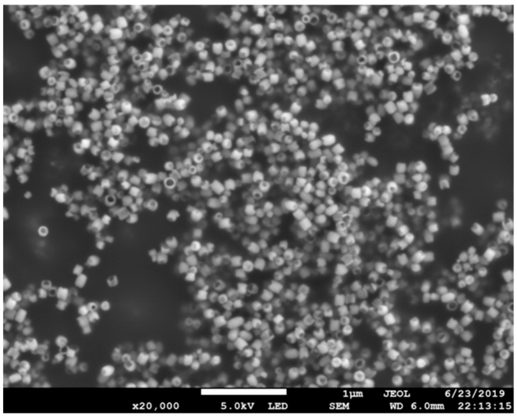

(**a**)

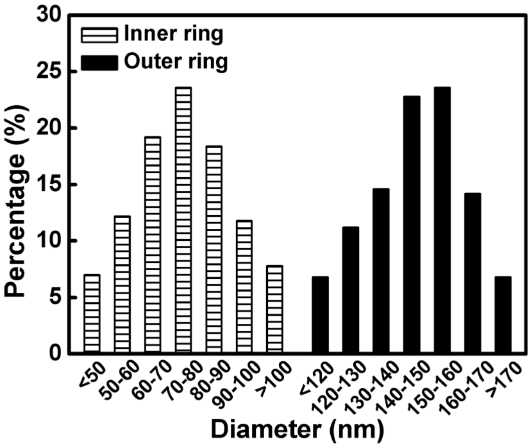

(**b**)

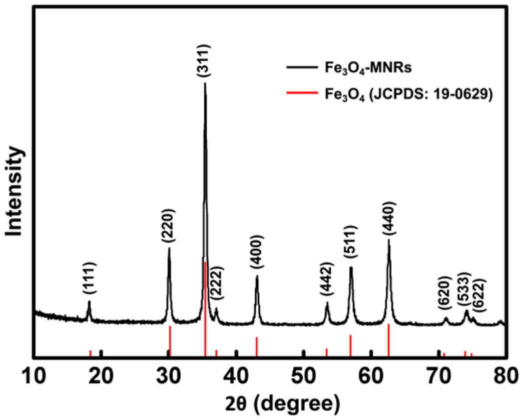

(**c**)

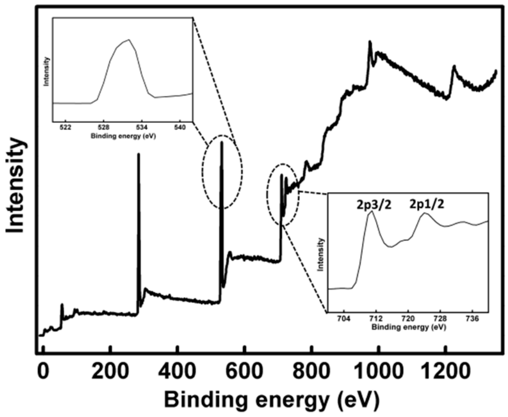

(**d**)

Figure S7. SEM image (**a**), size distribution (**b**), XRD pattern (**c**) and XPS spectra (**d**) of the as-prepared Fe_3O_4-MNRs after 10 cycles of use.

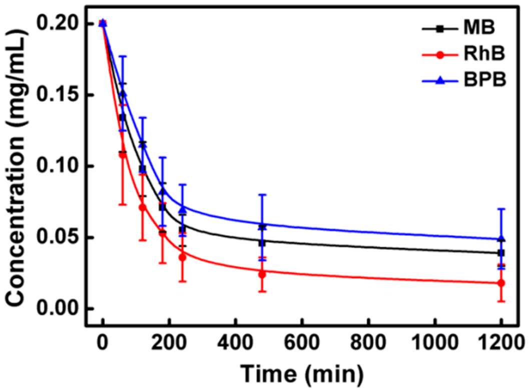

Figure S8. Kinetics of the degradation of dyes at three different reaction periods.

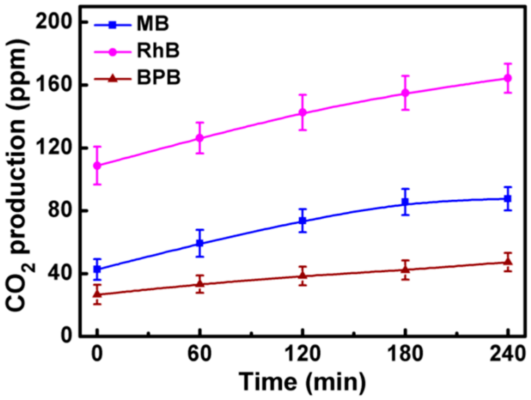

Figure S9. CO_2 production process of different dyes.

Table S1. Calculation of average crystallite size by using Scherrer equation.

B obs. [2Th]	B std. [2Th]	Pos. [2Th]	Crystallite size(Å)
0.354	0.060	30.194	280
0.394	0.060	35.593	250
0.315	0.060	43.170	335
0.315	0.060	53.644	349
0.472	0.060	57.171	219

Table S2. Kinetic parameters for dyes degradation by Fe_3O_4-MNRs with different concentration.

Fe_3O_4-MNRs dosage g/L	MB			BPB			RhB		
	R^2	q_m mg/g	k_2 g/(mg·h)	R^2	q_m mg/g	k_2 g/(mg·h)	R^2	q_m mg/g	k_2 g/(mg·h)
0.1	0.990	14.46	0.005	0.995	12.97	0.004	0.996	18.69	0.007
0.5	0.994	12.16	0.032	0.992	10.25	0.024	0.998	15.26	0.045
1.0	0.997	9.75	0.068	0.999	7.69	0.051	0.995	11.25	0.076
